# Association between N, N-diethyl-m-toluamide exposure and the odds of kidney stones in US adults: a population-based study

**DOI:** 10.3389/fpubh.2023.1248674

**Published:** 2023-11-24

**Authors:** Chengcheng Wei, Jiatai He, Zhuo Wei, Yu Huang, Ming Xiong, Changqi Deng, Zhaohui Chen, Wencheng Li, Xiaoping Zhang

**Affiliations:** ^1^Department of Urology, Union Hospital, Tongji Medical College, Huazhong University of Science and Technology, Wuhan, China; ^2^Department of Urology, The Central Hospital of Xiaogan, Xiaogan, China

**Keywords:** N, N-diethyl-m-toluamide, DEET exposure, kidney stones, NHANES survey, public health

## Abstract

**Background:**

Currently, there is limited research on the specific relationship between N, N-diethyl-m-toluamide (DEET) exposure and the odds of kidney stones. We aimed to investigate the relationship between DEET exposure and the prevalence of kidney stones.

**Methods:**

We included 7,567 qualified participants in our research from the 2007–2016 NHANES survey. We carried out three logistic regression models to explore the potential association between DEET exposure and the odds of kidney stones. Spline smoothing with generalized additive models (GAM) was utilized to assess the non-linear relationship and restricted cubic spline (RCS) curves was to determine the dose–response association. Multivariate regression models were used to conduct stratified analysis and sensitivity analysis.

**Results:**

Baseline characteristics of study participants presented the distribution of covariables. Regression analysis revealed that the odds of kidney stones were positively associated with the main metabolites of 3-diethyl-carbamoyl benzoic acid (DCBA) (log2) (OR = 1.05, 95% CI 1.02 to 1.08). The fourth quartile of urine DCBA showed a greater risk of kidney stones in the fully adjusted model (OR = 1.36, 95% CI 1.08 to 1.72). Another DEET metabolite of N, N-diethyl-3-hydroxymethylbenzamide (DHMB) was used to confirm the accuracy and stability of the results. The spline smoothing curve represented two main DEET metabolites had similar no-linear relationships and a positive trend with kidney stones proportion. RCS implied that the incidence of kidney stones rose with increasing levels of DEET exposure. High-risk groups on kidney stones were exhibited by stratified analysis under DEET exposure.

**Conclusion:**

Our study suggests that DEET exposure is positively associated with odds of kidney stones. Further investigation into the underlying processes of this association is required to guide the prevention and treatment of kidney stones.

## 1 Introduction

A global investigation of the incidence and epidemiology of kidney stones elucidates that a progressively worldwide growth has emerged in the past several decades, in not only developed but also developing countries, whether age, sexual distinction, or ethnicity differences (1, 2). In the United States, 1 out of 11 people suffer from kidney stones, and it is estimated that kidney stones affect 600,000 Americans annually (3, 4). However, within 5 to 10 years after the first treatment, no < 50% of the patients suffer from a higher relapse rate than other types of urologic diseases (5). More than that, kidney stone disease tends to become a systemic disease and develop other comorbidities such as arterial hypertension (6), metabolic bone disease (7), and chronic kidney disease (8). Obesity, diabetes, hypertension and some other noninfectious etiology, such as low fluid intake, are generally considered risk factors for stone formation (9, 10). Nonetheless, many inorganic compounds such as (phosphate, calcium oxalate) or organic substances (Nephrocalcin, Osteopontin) can also influence stone formation and being promotors or inhibitors (11). However, there is currently little research on the impact of organic substances on kidney stones. Therefore, focusing on common organic compounds that people are exposed to on a daily basis is another significant method for preventing and treating kidney stones.

As a broad-spectrum insect repellent, N, N-diethyl-meta-toluamide (DEET) has been widely pervasive since the early 1950s (12, 13). The estimated annual production of DEET in the United States is 1800 tons (14). However, DEET in the aquatic environment has been emerging pollutants (12), because of the difficulty in effectively eliminating it by conventional treatment processes (15), and the maximum detection concentration in European landfill leachate was as high as 320.00 μg/L (16). The main route of exposure to DEET for the general population is through dermal contact, and DEET is commonly absorbed through the skin and digestive tract (17). Tynaliev (18) have reported that toxic chemicals including pesticides are involved in kidney stone genesis by tubular impairment, and frequent occurrence of recurrent kidney stone in patients known to acquire high poisonous chemicals levels in renal tissue and urine. As one of the pesticides, DEET plays a vital role in causing human intoxication by decreasing the permeability of the blood-brain barrier (17). In addition, there is evidence that DEET can induce CYPs 3A4, 2B6, 2A6, 1A1, and 1A2 translation and transcription, thereby inducing its own metabolism *in vitro* human (19), which may be the foundation of mechanisms of toxicity of DEET. Besides, DEET has been proven to have sub-lethal effects on cardiovascular system (20) which frequently manifests as bradycardia and hypotension (21). Kidney stones were correlated with an elevated risk of cardiovascular disease. Consequently, like other toxic chemicals, DEET may promote the formation of kidney stones by directly targeting the kidney or indirectly acting through other systems (22).

Currently, some studies have discovered that exposure to particular chemicals, including pesticides and herbicides, may increase the odds of kidney stones (23–25). However, there is limited research on the specific relationship between DEET exposure and the odds of kidney stones. As a common pesticides and environmental pollutant, excreting through urine (26), DEET has the potential to cause the formation of kidney stones. Furthermore, as the main metabolites of DEET, DCBA is expected to become an effective biomarker of DEET because of its detectability, which may facilitate the following research (27). Thus, we hypothesize that DEET exposure may potentially increase the incidence of kidney stones. Our study aims to analyze the relationship between DEET exposure and the prevalence of kidney stones using a population-based study from the NHANES survey to validate the hypothesis.

## 2 Methods

### 2.1 Source of data

The National Health and Nutrition Examination Study (NHANES) is a countrywide study undertaken by the Centers for Disease Control and Prevention (CDC) to evaluate the health and nutritional status of the American population (28). Since the early 1960s, the NHANES program has been conducted as a series of surveys, focusing on different demographic groups or health-related issues. In order to meet new demands, the survey was converted into a continuous program in 1999, with a rotating emphasis on various health and nutritional indicators. The NHANES website has all population statistics and methodological information (www.cdc.gov/nchs/nhanes). The National Center and all participants have approved NHANES protocols for the Ethics Review Committee for Health Statistics Research.

### 2.2 Study population

**Figure 1:**
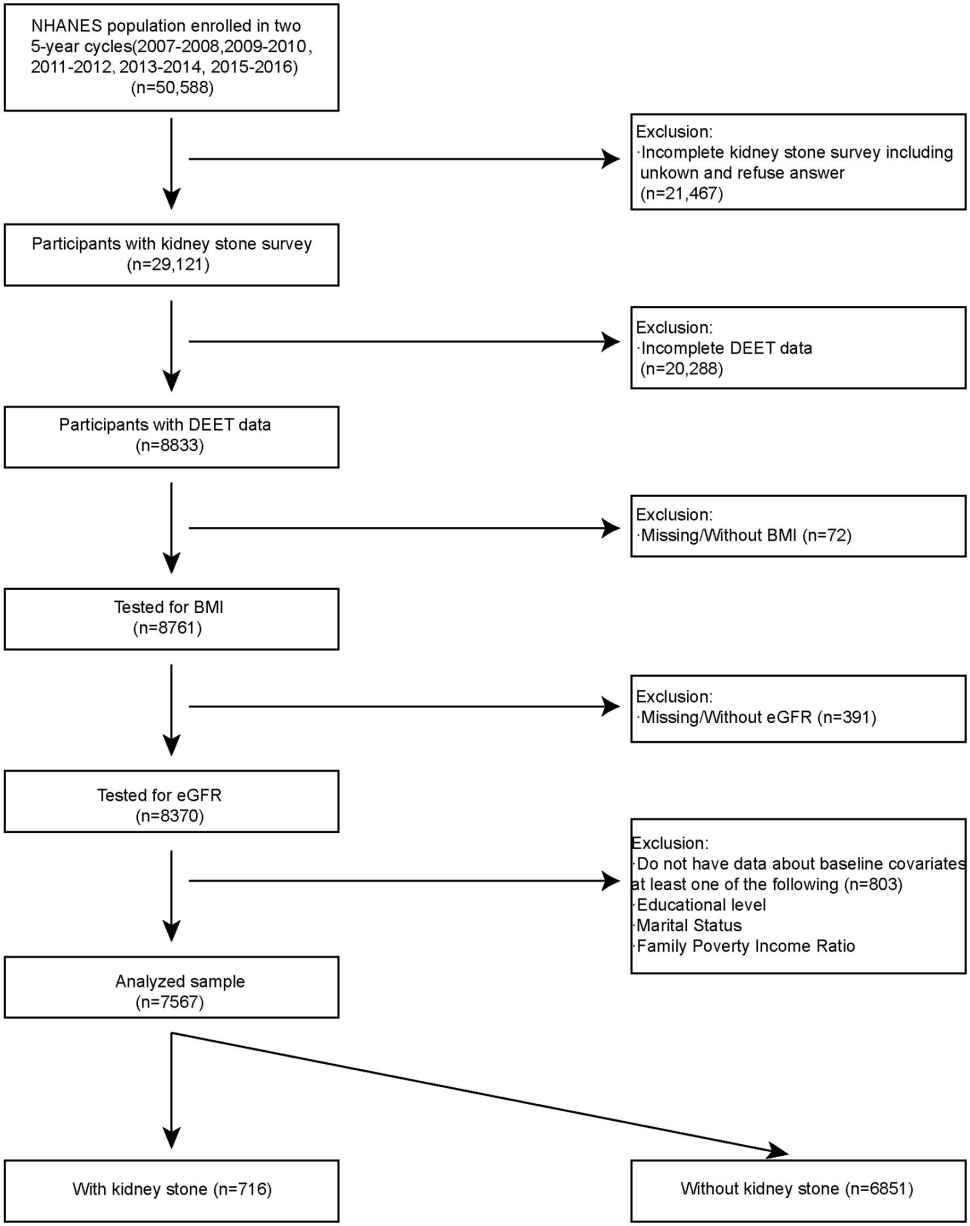
Flowchart for selecting the participants.

Using a complicated probability sampling methodology, standardized interviews, physical exams, and sampling tests, the NHANES data were collected from various groups and evaluated to determine the dietary and physical health status of non-hospitalized individuals in the USA. We included NHANES open data in five cycles for analysis (2007–2008, 2009–2010, 2011–2012, 2013–2014, 2015–2016). There are a total 50,588 of participants fitted into our research. Participants had to fulfill the following inclusion requirements in order to be chosen as eligible participants for analysis: (1) Participants with kidney stones survey (*n* = 29,121); (2) Participants with DEET data (8833); (3) Tested for BMI (*n* = 8,761); (4) Tested for baseline covariates (*n* = 8,370). A total of 7,567 participants were included in our analysis. Among them, 716 participants had kidney stones, and 6,851 participants did not have kidney stones (Figure 1).

### 2.3 Kidney stone status

Kidney stone status is extracted from “Kidney Conditions – Urology” in the NHANES Questionnaire Data part. Using the Computer-Assisted Personal Interview (CAPI) system (KIQ026), “Ever had kidney stones?” was asked in the home by trained interviewers. When participants answered “yes,” we identified that the participants had kidney stones in the past.

### 2.4 DEET measurement

For measuring DEET and its metabolites, such as N, N-diethyl-3-hydroxymethylbenzamide (DHMB) and 3-diethyl-carbamoyl benzoic acid (DCBA), in 100 L of human urine, this method uses online solid phase extraction in conjunction with high performance liquid chromatography-tandem mass spectrometry (SPE-HPLC-MS/MS). The first step in sample preparation is an overnight enzymatic deconjugation of the metabolites attached to glucuronides. The following day, the chemicals being tested are concentrated using online SPE, and using reversed phase HPLC, they are chromatographically separated from one another and from other urine biomolecules. Atmospheric Pressure Chemical Ionization (APCI) is used to transform the eluting molecular ions into gas phase ions, which are then specifically filtered by mass-to-charge ratios with unit resolution. The chosen molecule ions are then dissociated chemically (CID), and the resultant product ions are filtered at unit resolution before being detected by an electron multiplier. This value of DEET and metabolites is the lower limit of detection divided by the square root of 2 (LLOD/sqrt [2]).

### 2.5 Covariates

We included factors that could affect the levels of kidney stones (25, 29, 30). In more detail, sociodemographic variables included age (in years), the ratio of poverty to income, race or ethnicity, level of education, and marital status. Body mass index (Kg/m^2^) is a piece of information from a medical exam and a person's life history into our research. Also, drinking status refers to those who had at least 12 drinks of alcohol in the past year, and smoking status refers to those who smoked at least 100 cigarettes in their lifetime. Some of the comorbidities included diabetes (yes/no) and hypertension (yes/no). Last, we estimated glomerular filtration rate (eGFR) using the Chronic Kidney Disease Epidemiology Collaboration equation (31): Male: GFR = 141 × min (Scr/0.9, 1)– 0.411 × max (Scr/0.9, 1)−1.209 × 0.993 Age × 1.159 (if black); Female: GFR = 141 × min (Scr/0.7, 1) – 0.329 × max (Scr/0.7, 1)−1.209 × 0.993 Age × 1.018 × 1.159 (if black).

### 2.6 Statistical analysis

Our research was based on the CDC guidelines' criteria, which were presented at (https://www.cdc.gov/nchs/nhanes/index.htm), and analysis was referred from the NHANES analytic guidelines (https://wwwn.cdc.gov/nchs/nhanes/tutorials/default2.aspx). We used the stratum and PSU variables (SDMVSTRA and SDMVPSU, respectively) from the demographic data file and the subsample weights from the DEET sample (WTSB2YR) for weighted sampling. Continuous variables were represented by the mean and standard deviation (SD) for the baseline characteristics, while categorical variables were shown as proportions or frequencies. We utilized three logistic regression models to investigate the potential link between N, N-diethyl-m-toluamide (DEET) exposure and the risk of kidney stones. In the non-adjusted model, we adjust for none. In the Minimally adjusted model, we adjust for gender, age, race, education, and marital status. Also, we include gender, age, race, education, marital status, PIR, BMI, drinking, smoking, HBP, diabetes, and glomerular filtration rate (eGFR) in the fully adjusted model. Additionally, a logistic regression model was fitted in order to determine the *P*-value for trend. The subgroup connection among urine DCBA and kidney stones was further investigated using the same methodology. After that, we used spline smoothing with a generalized additive model (GAM) to examine if there was a non-linear correlation between DEET exposure and kidney stones. We employed a restricted cubic spline function to clarify the dose-response association between DEET exposure and the incidence of kidney stones based on the fully adjusted model. We also conducted a series of sensitivity analyses to test the robustness of our results. First, because participants exposure to DEET work environment could cause extreme data, we deleted extreme DCBA and DHMB values (< 5 and > 95%). Then, we constructed multivariate regression models to conduct sensitivity analysis. R (http://www.R-project.org; The R Foundation) was used to carry out every analysis. *P* < 0.05 was regarded as statistically significant on both sides.

## 3 Results

### 3.1 Baseline characteristics of research participants

Figure 1 listed the criteria for inclusion and exclusion, and we have identified 7,567 qualified participants out of 50,588 participants who fit into our research from the 2007–2016 NHANES survey. Urine 3-diethyl-carbamoyl benzoic acid (DCBA) was the main metabolite of DEET. Table 1 displayed the initial clinical characteristics of selected participants according to the DCBA content categories. We grouped the population by quartile according to the DCBA (Q1-Q4). We found significant differences between the DEET exposure and several variables, including sex, age, gender, race, education, marital status, BMI, diabetes, HBP, and glomerular filtration rate (eGFR), with p < 0.05. The table showed that younger participants had higher urinary DCBA concentrations [Q1:51.66 ± 17.24 (years), Q2:48.99 ± 17.66 (years), Q3: 47.76 ± 17.51 (years), Q4: 47.54 ± 17.24 (years)]. And compared with females, more males suffered higher DEET exposure [For male, Q1:839 (44.34%), Q2: 891 (47.19%), Q3: 891 (47.19%), Q4: 1,011 (53.44%); For female, Q1: 1,053 (55.66%), Q2: 997 (52.81%), Q3: 961 (50.71%), Q4: 881 (46.56%)]. Then, we constructed the population's characteristics based on the types of kidney stones (Table 2). Among them, there were a total 716 participants with kidney stones and 6,851 cases without stone formation. Compared with people no kidney stone formation, we found people with kidney stones had higher DEET exposure, including DEET, DCBA, and N, N-diethyl-3-hydroxymethylbenzamide (DHMB). Kidney stone participants with no kidney stone participants, DEET concentration was DEET (ug/L) (mean ± SD) (0.10 ± 1.63 compared with 0.08 ± 0.19, *P* = 0.001); DCBA concentration was DCBA (ug/L) (mean ± SD) (106.65 ± 4689.81 compared with 108.00 ± 1217.17, *P* = 0.01); DHMB concentration was DHMB (ug/L) (mean ± SD) (2.59 ± 153.47 compared with 0.56 ± 4.76, *P* = 0.009).

**Table 1:** Characteristics of participants by DCBA content categories: NHANES 2007–2016.

	**Q1**	**Q2**	**Q3**	**Q4**	***P*-value**
***N***	1,892	1,888	1,895	1,892	
**Age**	51.66 ± 17.24	48.99 ± 17.66	47.76 ± 17.51	47.54 ± 17.24	< 0.001
**Gender (** ***N*** **, %)**					< 0.001
Male	839 (44.34)	891 (47.19)	934 (49.29)	1,011 (53.44)	
Female	1,053 (55.66)	997 (52.81)	961 (50.71)	881 (46.56)	
**Race (** ***N*** **, %)**					< 0.001
Mexican American	305 (16.12)	264 (13.98)	266 (14.04)	288 (15.22)	
Other Hispanic	192 (10.15)	205 (10.86)	214 (11.29)	161 (8.51)	
Non-Hispanic white	807 (42.65)	805 (42.64)	793 (41.85)	893 (47.20)	
Non-Hispanic black	248 (13.11)	429 (22.72)	463 (24.43)	407 (21.51)	
Other races	340 (17.97)	185 (9.80)	159 (8.39)	143 (7.56)	
**Education (** ***N*** **, %)**					< 0.001
Less than college	814 (43.02)	852 (45.13)	875 (46.17)	929 (49.10)	
Some college	542 (28.65)	573 (30.35)	613 (32.35)	553 (29.23)	
College graduate or above	536 (28.33)	463 (24.52)	407 (21.48)	410 (21.67)	
**Marital status (** ***N*** **, %)**					< 0.001
Married	1,064 (56.24)	942 (49.89)	906 (47.81)	1,028 (54.33)	
Single	733 (38.74)	799 (42.32)	835 (44.06)	694 (36.68)	
Living with a partner	95 (5.02)	147 (7.79)	154 (8.13)	170 (8.99)	
**Poverty income ratio (mean** **±SD)**	2.70 ± 1.66	2.47 ± 1.61	2.38 ± 1.62	2.44 ± 1.63	< 0.001
**Body mass index (kg/m2) (mean** **±SD)**	28.29 ± 6.41	29.11 ± 6.90	29.59 ± 7.13	29.65 ± 7.04	< 0.001
**Smoking (** ***N*** **, %)**					< 0.001
< 100 cigarettes in life	761 (40.22)	798 (42.27)	860 (45.38)	971 (51.32)	
≥100 cigarettes in life	1,130 (59.73)	1,090 (57.73)	1,034 (54.56)	920 (48.63)	
**Alcohol (** ***N*** **, %)**					< 0.001
< 12 drinks/year	1,194 (68.50)	1,242 (71.09)	1,270 (72.41)	1,342 (76.34)	
≥12 drinks/year	549 (31.50)	503 (28.79)	483 (27.54)	415 (23.61)	
**Diabetes (** ***N*** **, %)**					0.840
Yes	238 (12.58)	230 (12.18)	219 (11.56)	211 (11.15)	
No	1,614 (85.31)	1,618 (85.70)	1,630 (86.02)	1,637 (86.52)	
**Hypertension (** ***N*** **, %)**					0.864
Yes	685 (36.21)	668 (35.38)	688 (36.31)	671 (35.47)	
No	1,206 (63.74)	1,217 (64.46)	1,206 (63.64)	1,218 (64.38)	
**Kidney stones (** ***N*** **, %)**					0.043
Yes	1,735 (91.70)	1,708 (90.47)	1,723 (90.92)	1,685 (89.06)	
No	157 (8.30)	180 (9.53)	172 (9.08)	207 (10.94)	
**Glomerular filtration rate (eGFR) ml/min (mean** **±SD)**	97.05 ± 12.42	99.66 ± 12.60	100.53 ± 12.85	100.87 ± 12.64	< 0.001
**DEET (ug/L) (mean** **±SD)**	0.06 ± 0.02	0.06 ± 0.02	0.06 ± 0.04	0.23 ± 3.16	< 0.001
**DCBA (ug/L) (mean** **±SD)**	0.45 ± 0.17	1.27 ± 0.39	3.95 ± 1.45	421.37 ± 8949.91	< 0.001
**DHMB (ug/L) (mean** **±SD)**	0.06 ± 0.01	0.06 ± 0.02	0.07 ± 0.08	9.80 ± 298.29	< 0.001

Q1–Q4: Grouped by quartile according to the DCBA (Q1-Q4). If it is a continuous variable, the Kruskal–Wallis rank sum test was used to determine it. The p-value for continuous variables with a theoretical value of < 10 was determined using Fisher's exact probability test. With regard to categorical data, the p-value was calculated using weighted chi-square. Values are presented as means + SD or %. SD, standards deviation; DEET, N, N-diethyl-m-toluamide; DCBA, 3-diethyl-carbamoyl benzoic acid; DHMB N, N-diethyl-3-hydroxymethylbenzamide.

**Table 2:** Baseline characteristics of selected participants in NHANES between 2007 and 2016 by categories of kidney stones.

	**All**	**Without kidney stones (*n =* 6,851)**	**With kidney stones (*n =* 716)**	
**Age**	48.99 ± 17.49	48.31 ± 17.46	55.44 ± 16.41	< 0.001
**Gender (** ***N*** **, %)**				< 0.001
Male	3,675 (48.57)	3,284 (47.93)	391 (54.61)	
Female	3,892 (51.43)	3,567 (52.07)	325 (45.39)	
**Race (** ***N*** **, %)**				< 0.001
Mexican American	1,123 (14.84)	1,019 (14.87)	104 (14.53)	
Other Hispanic	772 (10.20)	692 (10.10)	80 (11.17)	
Non-Hispanic white	3,298 (43.58)	2,917 (42.58)	381 (53.21)	
Non-Hispanic black	1,547 (20.44)	1,447 (21.12)	100 (13.97)	
Other races	827 (10.93)	776 (11.33)	51 (7.12)	
**Education (** ***N*** **, %)**				0.013
Less than college	3,470 (45.86)	3,118 (45.51)	352 (49.16)	
College	2,281 (30.14)	2,057 (30.02)	224 (31.28)	
College graduate or above	1,816 (24.00)	1,676 (24.46)	140 (19.55)	
**Marital status (** ***N*** **, %)**				< 0.001
Married	3,940 (52.07)	3,510 (51.23)	430 (60.06)	
Single	3,061 (40.45)	2,808 (40.99)	253 (35.34)	
Living with a partner	566 (7.48)	533 (7.78)	33 (4.61)	
**Poverty income ratio (mean** **±SD)**	2.50 ± 1.63	2.50 ± 1.63	2.47 ± 1.61	0.601
**Body mass index (kg/m**^**2**^**) (mean** **±SD)**	29.16 ± 6.89	28.99 ± 6.89	30.78 ± 6.73	< 0.001
**Smoking (** ***N*** **, %)**				0.025
< 100 cigarettes in life	3,390 (44.80)	3,031 (44.24)	359 (50.14)	
≥100 cigarettes in life	4,174 (55.16)	3,817 (55.71)	357 (49.86)	
**Alcohol (** ***N*** **, %)**				0.397
< 12 drinks/year	5,048 (72.09)	4,575 (72.30)	473 (70.18)	
≥12 drinks/year	1,950 (27.85)	1,749 (27.64)	201 (29.82)	
**Diabetes (** ***N*** **, %)**				< 0.001
Yes	898 (11.87)	737 (10.76)	161 (22.49)	
No	6,499 (85.89)	5,973 (87.18)	526 (73.46)	
**Hypertension (** ***N*** **, %)**				< 0.001
Yes	2,712 (35.84)	2,339 (34.14)	373 (52.09)	
No	4,847 (64.05)	4,504 (65.74)	343 (47.91)	
**Glomerular filtration rate (eGFR) ml/min (mean** **±SD)**	99.53 ± 12.72	99.96 ± 12.72	95.39 ± 11.96	< 0.001
**DEET (ug/L) (mean** **±SD)**	0.10 ± 1.55	0.10 ± 1.63	0.08 ± 0.19	0.001
**DCBA (ug/L) (mean** **±SD)**	106.78 ± 4478.05	106.65 ± 4689.81	108.00 ± 1217.17	0.01
**DHMB (ug/L) (mean** **±SD)**	2.41 ± 146.30	2.59 ± 153.47	0.56 ± 4.76	0.009

If it is a continuous variable, the Kruskal–Wallis rank sum test was used to determine it. The p-value for continuous variables with a theoretical value of < 10 was determined using Fisher's exact probability test. With regard to categorical data, the p-value was calculated using weighted chi-square. Values are presented as means + SD or %. SD, standards deviation; DEET, N, N-diethyl-m-toluamide; DCBA, 3-diethyl-carbamoyl benzoic acid; DHMB N, N-diethyl-3-hydroxymethylbenzamide.

### 3.2 Multivariate regression analysis

We have constructed three models to better understand the potential link between DEET exposure and the odds of kidney stones. For the independent DEET and its metabolites, we utilized five statistical tests: the Anderson-Darling normality test, the Cramer-von Mises normality test, the Lilliefors (Kolmogorov-Smirnov) normality test, the Pearson chi-square normality test, and the Shapiro–Francia normality test to identify the normality of the inflammation-related index distribution (Supplementary Table S1), and we used log2-transformed to improve the normal distribution. Urinary DCBA was the main metabolite of DEET; thus, we first analyzed urinary DCBA and the odds of kidney stones. We found the odds of kidney stones were positively associated with the urine DCBA (log2) in three models (OR = 1.05, 95% CI 1.02 to 1.08). Then we categorized the participants by the quartile of DCBA. A positive correlation was showed between the occurrence of kidney stones and the rising concentration of urine DCBA, as evidenced by the results of trend test analysis. In all three models, the *P*-value was discovered to be < 0.05. In particular, the fourth quartile of urine DCBA concentration had higher odds of kidney stones compared to the first quartile, with a significant increase in odds ratios in the non-adjusted model (OR = 1.36, 95% CI 1.09 to 1.69, *P* = 0.006), the minimally adjusted model (OR = 1.42, 95% CI 1.14 to 1.78, *P* = 0.0019), and the fully adjusted model (OR = 1.36, 95% CI 1.08 to 1.72, *P* = 0.0095). We also constructed a multivariate logistic regression model between another DEET metabolite of urine DHMB and odds of kidney stones to validate the reliability and stability of the findings. The prevalence of kidney stones was positively associated with the urine DHMB (log2) in three models. In the non-adjusted model (OR = 1.08, 95% CI 1.02 to 1.14, *P* = 0.0071), in the minimally adjusted model (OR = 1.07, 95% CI 1.01 to 1.13, *P* = 0.019), in the fully adjusted model (OR = 1.08, 95% CI 1.021 to 1.14, *P* = 0.0126). Based on the DHMB distribution, we categorized the participants by 90% double with T1 and T2. Compared to the T1, the T2 of urine DHMB had higher odds of kidney stone, with a significant increase in odds ratios in the non-adjusted model (OR = 1.35, 95% CI 1.03 to 1.76, *P* = 0.0269) and the fully adjusted model (OR = 1.34, 95% CI 1.01 to 1.76, *P* = 0.0397). We also observed P for trend with statistical differences on the positive correlation in two models. In all, higher level of DEET exposure was more susceptible to kidney stones (Table 3).

**Table 3:** Multivariate-adjusted odds of urine DCBA or DHMB in all participants and the association between the quartile of urine DCBA or DHMB and kidney stones.

**Exposure**	***N***	**Non-adjusted model** ^*^		**Minimally adjusted model** ^**^		**Fully adjusted model** ^***^	
		**OR (95% CI)**	***P*** **-value**	**OR (95% CI)**	***P*** **-value**	**OR (95% CI)**	***P-*** **value**
DCBA-log2 (ug/L)		1.05 (1.02, 1.08)	0.0013	1.05 (1.02, 1.08)	0.0009	1.05 (1.02, 1.08)	0.0037
Q1	1,892	Reference		Reference		Reference	
Q2	1,888	1.16 (0.93, 1.46)	0.1828	1.23 (0.98, 1.55)	0.0747	1.23 (0.97, 1.56)	0.0873
Q3	1,895	1.10 (0.88, 1.38)	0.3953	1.18 (0.94, 1.49)	0.1539	1.17 (0.92, 1.48)	0.2037
Q4	1,892	1.36 (1.09, 1.69)	0.006	1.42 (1.14, 1.78)	0.0019	1.36 (1.08, 1.72)	0.0095
P for trend		0.013		0.005		0.021	
DHMB-log2 (ug/L)		1.08 (1.02, 1.14)	0.0071	1.07 (1.01, 1.13)	0.019	1.08 (1.02, 1.14)	0.0126
T1	5,508	Reference		Reference		Reference	
T2	613	1.35 (1.03, 1.76)	0.0269	1.30 (0.99, 1.70)	0.0553	1.34 (1.01, 1.76)	0.0397
P for trend		0.027		0.058		0.043	

CI, confidence interval; OR, odds ratio. ^*^The non-adjusted model adjusts for none. ^**^The minimally adjusted model adjusts for gender, age, race, education, and marital status. ^***^The fully adjusted model adjusts for gender, age, race, education, marital status, poverty income ratio (PIR), BMI, smoking, alcohol drinking, hypertension, diabetes, glomerular filtration rate (eGFR). Q1: 0.1414–0.6865 (ug/L); Q2: 0.6865–2.05 (ug/L); Q3: 2.05–7.315 (ug/L); Q4: 7.315–382000 (ug/L). T1: 0.0629–0.157 (ug/L); T2: 0.157–11400 (ug/L).

### 3.3 Spline smoothing and restricted cubic spline between DEET exposure and the kidney stones

**Figure 2:**
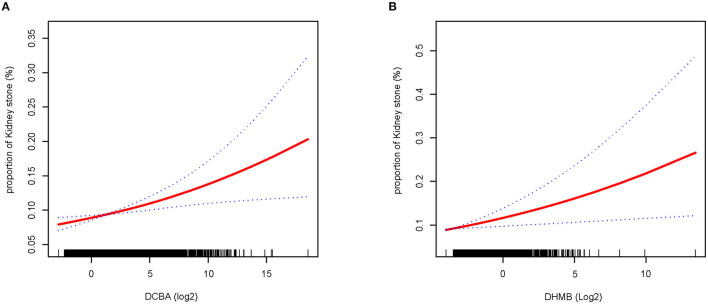
Smooth fit curves reveal the non-linear relationship between DEET exposure and the proportion of nephrolithiasis. **(A)** Relationship between DCBA and kidney stones **(B)** relationship between DHMB and kidney stones; Model adjusts for gender, age, ethnicity, education, marital status, poverty income ratio (PIR), BMI, smoking, alcohol drinking, high blood pressure, diabetes, glomerular filtration rate (eGFR).

**Figure 3:**
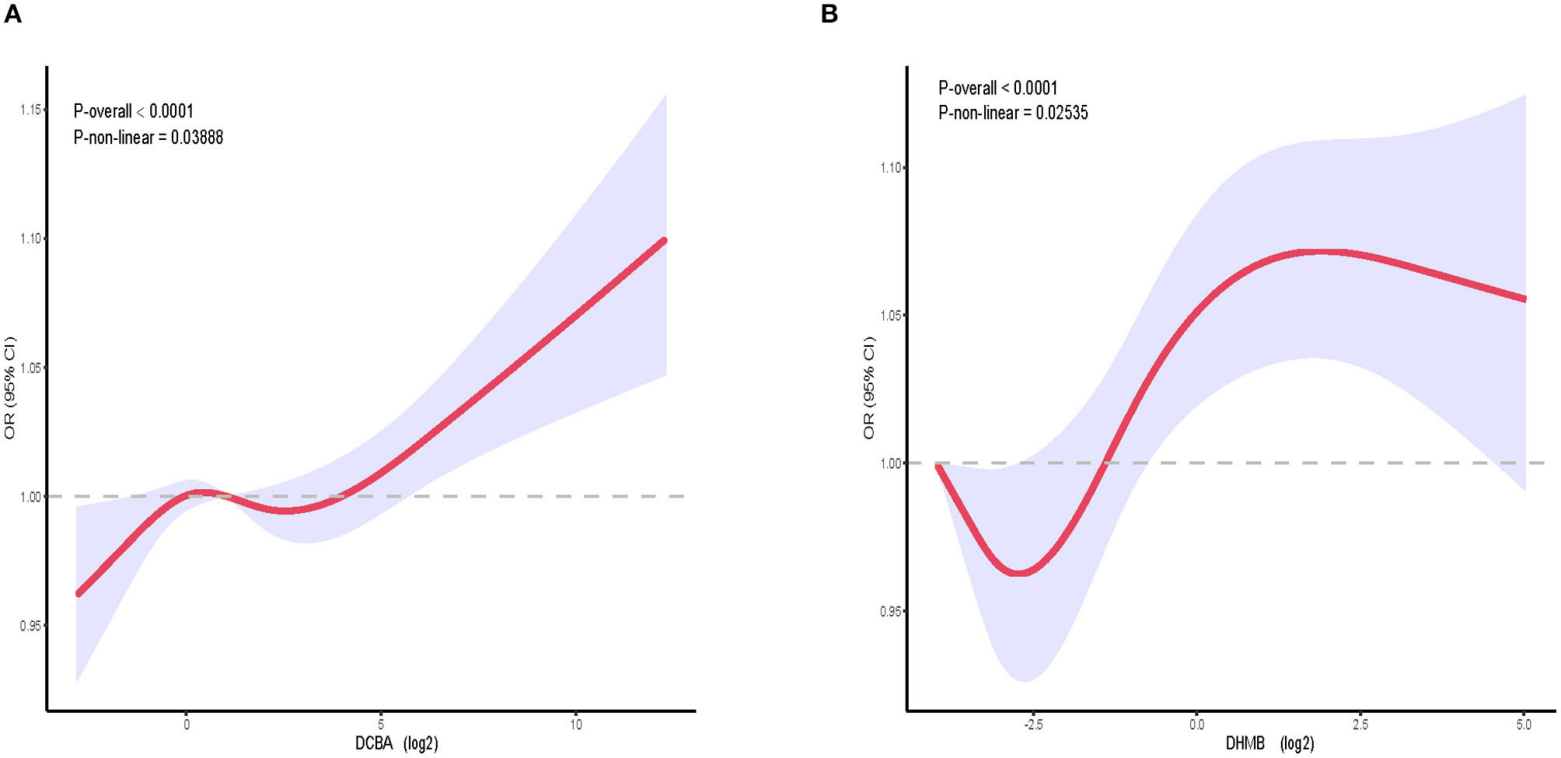
The relative risk of kidney stones in the general population dependent on DEET exposure. OR, based on restricted cubic splines of DEET exposure level, is represented by solid lines. The upper and lower 95% CI bounds are shaded. The adjustment factors are identical to those in the expanded model. **(A)** The connection between DCBA and kidney stones. **(B)** The connection between DHMB and kidney stones; Model adjusts for gender, age, ethnicity, education, marital status, poverty income ratio (PIR), BMI, smoking, alcohol drinking, high blood pressure, diabetes, glomerular filtration rate (eGFR).

We constructed a spline smoothing curve to investigate the non-linear relationship between DEET exposure and kidney stones (Figure 2), which controls for gender, age, ethnicity, education, marital status, poverty income ratio (PIR), BMI, smoking, alcohol, high blood pressure, diabetes, glomerular filtration rate (eGFR). From this curve, we observed a non-linear association between DEET exposure in DCBA (log2) and DHMB (log2) and the occurrence of kidney stones. Higher DEET exposure was related to more kidney stones, which indicated a positive connection. When the urine DEET metabolite increased, the gradient of kidney stone proportion rose. We additionally conducted a dose-response analysis curves of restricted cubic spline function, which revealed that rising levels of DEET exposure increased the likelihood of kidney stones forming (Figure 3).

### 3.4 Subgroup analysis

We applied stratified models to determine the high-risk population for DEET exposure and kidney stones, which is shown in Table 4. We found the odds of kidney stones was positively associated with the urine DCBA (log2) in female group (OR =1.06, 95% CI 1.01 to 1.11), age < 60 group (OR =1.09, 95% CI 1.05 to 1.13), race of Mexican American group (OR =1.11, 95% CI 1.02 to 1.21), race of non-Hispanic white group (OR = 1.05, 95% CI 1.01 to 1.10), education of college graduate or above group (OR =1.07, 95% CI 1.00 to 1.14), marital status of single group (OR = 1.09, 95% CI 1.03 to 1.14), BMI ≥25 group (OR = 1.05, 95% CI 1.02 to 1.09), no diabetes group (OR =1.06, 95% CI 1.02 to 1.09), no hypertension group (OR = 1.07, 95% CI 1.03 to 1.11), Glomerular filtration rate (eGFR) ≥90 group (OR = 1.09, 95% CI 1.05 to 1.12) with statistical differences. We also discovered that the probability of developing kidney stones increased with the Q4 level of urine DCBA in the above subgroups. In the female group (OR = 1.51, 95% CI 1.07 to 2.13), age < 60 group (OR = 1.72, 95% CI 1.24 to 2.39), marital status of the single group (OR = 1.98, 95% CI 1.33 to 2.95), BMI ≥25 group (OR = 1.34, 95% CI 1.03 to 1.74), no diabetes group (OR = 1.31, 95% CI 1.01 to 1.71), no hypertension group (OR = 1.55, 95% CI 1.11 to 2.18), glomerular filtration rate (eGFR) ≥90 group (OR = 1.72, 95% CI 1.27 to 2.32). In all, we identified high-risk groups for kidney stones under DEET exposure through subgroup analysis.

**Table 4:** Stratified analysis of urine DCBA odds in subgroup individuals and the relationship between the urine DCBA quartile and kidney stones.

	**DCBA (Log2)**	**Q1**	**Q2**	**Q3**	**Q4**
**Gender**					
Male	1.04 (1.00, 1.08) 0.0616	Reference	1.27 (0.91, 1.76) 0.1572	1.22 (0.88, 1.69) 0.2425	1.27 (0.92, 1.76) 0.1418
Female	1.06 (1.01, 1.11) 0.0114	Reference	1.22 (0.86, 1.73) 0.2556	1.08 (0.75, 1.54) 0.6913	**1.51 (1.07, 2.13) 0.0189**
**Age**					
< 60	1.09 (1.05, 1.13) < 0.0001	Reference	1.36 (0.96, 1.92) 0.0793	1.22 (0.86, 1.72) 0.2644	**1.72 (1.24, 2.39) 0.0012**
≥60	0.99 (0.95, 1.04) 0.7582	Reference	1.12 (0.80, 1.57) 0.4943	1.18 (0.84, 1.65) 0.3474	1.02 (0.72, 1.45) 0.8996
**Race**					
Mexican American	1.11 (1.02, 1.21) 0.0114	Reference	0.62 (0.31, 1.26) 0.1870	1.05 (0.57, 1.93) 0.8712	1.49 (0.85, 2.63) 0.1673
Other Hispanic	1.04 (0.94, 1.15) 0.4554	Reference	1.06 (0.53, 2.11) 0.8681	0.74 (0.36, 1.51) 0.4074	1.17 (0.56, 2.46) 0.6774
Non-Hispanic white	1.05 (1.01, 1.10) 0.0124	Reference	1.11 (0.80, 1.55) 0.5248	1.23 (0.88, 1.71) 0.2207	1.37 (0.99, 1.88) 0.0547
Non-Hispanic black	0.99 (0.90, 1.09) 0.8572	Reference	2.21 (1.08, 4.54) 0.0307	1.34 (0.62, 2.90) 0.4553	1.66 (0.78, 3.51) 0.1876
Other races	1.00 (0.88, 1.13) 0.9946	Reference	**2.89 (1.21, 6.88) 0.0166**	2.41 (0.97, 6.02) 0.0588	0.89 (0.28, 2.82) 0.8379
**Education**					
Less than college	1.04 (1.00, 1.09) 0.0584	Reference	1.19 (0.84, 1.68) 0.3395	1.19 (0.84, 1.70) 0.3293	1.37 (0.97, 1.93) 0.0699
College	1.03 (0.98, 1.09) 0.2580	Reference	1.14 (0.75, 1.72) 0.5526	0.99 (0.65, 1.50) 0.9460	1.24 (0.82, 1.88) 0.3161
College graduate or above	1.07 (1.00, 1.14) 0.0456	Reference	1.42 (0.84, 2.39) 0.1915	1.41 (0.82, 2.40) 0.2132	1.46 (0.86, 2.48) 0.1599
**Marital status**					
Married	1.04 (1.00, 1.08) 0.0846	Reference	1.20 (0.89, 1.62) 0.2384	1.15 (0.84, 1.56) 0.3791	1.13 (0.84, 1.52) 0.4336
Single	1.09 (1.03, 1.14) 0.0013	Reference	1.28 (0.85, 1.92) 0.2390	1.25 (0.83, 1.88) 0.2770	**1.98 (1.33, 2.95) 0.0008**
Living with a partner	0.93 (0.79, 1.10) 0.4000	Reference	1.37 (0.36, 5.25) 0.6489	1.23 (0.31, 4.97) 0.7684	1.31 (0.34, 5.00) 0.6962
**BMI**					
< 25	1.02 (0.95, 1.10) 0.5320	Reference	1.29 (0.76, 2.18) 0.3397	0.92 (0.51, 1.65) 0.7703	1.42 (0.83, 2.41) 0.2013
≥25	1.05 (1.02, 1.09) 0.0036	Reference	1.22 (0.93, 1.59) 0.1527	1.21 (0.93, 1.58) 0.1571	**1.34 (1.03, 1.74) 0.0272**
**Diabetes**					
Yes	1.06 (0.99, 1.13) 0.1082	Reference	1.58 (0.94, 2.67) 0.0835	1.42 (0.83, 2.41) 0.1998	1.60 (0.95, 2.71) 0.0791
No	1.06 (1.02, 1.09) 0.0008	Reference	1.15 (0.88, 1.52) 0.3044	1.09 (0.83, 1.44) 0.5365	**1.31 (1.01, 1.71) 0.0449**
**Hypertension**					
Yes	1.02 (0.97, 1.06) 0.4068	Reference	1.20 (0.87, 1.65) 0.2749	1.07 (0.77, 1.49) 0.6951	1.18 (0.85, 1.63) 0.3168
No	1.07 (1.03, 1.11) 0.0016	Reference	1.29 (0.91, 1.83) 0.1470	1.26 (0.89, 1.79) 0.1892	**1.55 (1.11, 2.18) 0.0107**
**Glomerular filtration rate (eGFR)**					
< 90	1.00 (0.95, 1.05) 0.9145	Reference	1.11 (0.77, 1.60) 0.5853	0.98 (0.67, 1.44) 0.9328	0.93 (0.63, 1.38) 0.7186
≥90	1.09 (1.05, 1.12) < 0.0001	Reference	1.36 (1.00, 1.86) 0.0537	1.35 (0.98, 1.84) 0.0626	**1.72 (1.27, 2.32) 0.0004**

Stratified models control for gender, age, ethnicity, education, marital status, Poverty income ratio (PIR), BMI, smoking, alcohol drinking; HBP, diabetes; eGFR, Glomerular filtration rate. The model was not modified for the stratification variable in any of the cases.

Bold values means statistical difference.

### 3.5 Sensitivity analysis

The results of the sensitivity analysis were presented in Supplementary Table S2. We constructed a multivariate regression model to conduct sensitivity analysis after deleting extreme DCBA and DHMB values. All results indicated that DEET exposure was positively correlated with the odds of kidney stones in the three models, which was consistent with the above findings.

## 4 Discussion

So far as we are aware, this is the first comprehensive analysis of the association between DEET exposure and the prevalence of kidney stones, based on data from five successive NHANES 2-year cycles (2007–2016) from a nationally representative population, despite the fact that it is a cross-sectional rather than a prospective study. Our findings revealed that DEET exposure was shown to be positively linked with the incidence of kidney stones, with the upper quartile having a greater risk of kidney stones than the lower quartile, no matter if adjusting for baseline characteristics including gender, age, race, education, marital status, etc.

With increasing incidence and recrudescence, kidney stones' pathogenesis is still unclear, although immune and inflammatory reactions, intestinal flora, and dietary regulation have been demonstrated to be dramatically correlated to stone formation (32–34). However, the significance of environmental factors and poor pollution control in the formation and progression of kidney stones has lately received a lot of attention (35, 36). N, N-Diethyl-m-toluamide (DEET) is largely used as a topical insect repellent against mosquitoes, ticks, and fleas, which has a great potential risk to environment protection and human health because of its wide spread consumer use, stable properties, and difficulty in degrading (21, 37, 38). In animals, DEET is swiftly absorbed from the skin, rapidly cleared from the circulation, rapidly metabolized in the liver, and promptly eliminated in the urine; Thus, in the metabolism process of DEET, there are quite a few organs including the kidney may suffer injuries and appear to have corresponding symptoms, including skin lesions, gait disturbance, grossly distended, and urine-filled bladders (21). Therefore, further research into the relationship between DEET exposure and kidney stones is required, considering there is little evidence about it.

Our finding that the occurrence of kidney stones was positively related to the DCBA in the non-adjusted model, the minimally adjusted model, and the fully adjusted model, which is also the main metabolite of DEET. Kuklenyik et al. (27) reported that DCBA was detected most frequently and at the highest concentrations, indicating that this may be a useful biomarker of DEET exposure (27). In addition, when we categorized the participants by the quartile of urine DCBA, we found that when compared to the first quartile of urine DCBA concentration, the fourth quartile exhibited a greater incidence of kidney stones, with a significant increase in odds ratios in the fully adjusted model (OR = 1.36, 95% CI 1.08 to 1.72, *P* = 0.0095) (Table 3). Furthermore, by constructing smooth fit curves between the DEET metabolite and the proportion of kidney stones, it was discovered that the gradient of kidney stone percentage increased as urine DEET metabolite levels rose (Figure 2). Dose-response analysis curves using a restricted cubic spline function revealed that rising levels of DEET exposure increased the chance of kidney stones forming (Figure 3). Finally, a higher DCBA content in urine made it more susceptible to kidney stones. In addition, in the subgroup analysis, we identified the high-risk group for kidney stones under DEET exposure, and many studies have indicated that the prevalence of kidney stones rose as BMI quartile increased (39–41), which may explained why higher BMI has been linked to an increase in filtration fraction due to renal hemodynamic alteration in heavier individuals (42, 43). It is also reported that female rats and young animals were more sensitive and susceptible to the effects of DEET toxicity than males or adult animals in animal studies (44), which is consistent with our results.

Kidney stones are increasingly considered a systematic and comprehensive disease, with a high risk of developing hypertension, diabetes, metabolic syndrome, chronic kidney disease and kidney failure (33, 45, 46). Currently, DEET exposure can damage multiple organ systems and cause mild or severe clinical manifestations, including seizures, coma, bradycardia, abdominal pain, skin irritation, and urticaria or contact rash (21, 37), it even has carcinogenic potential in human nasal mucosal cells (47), which is similar to another environmental pollutant ethylene oxide (46, 47). Importantly, when rats were given DEET in corn oil via gavage, the liver, lung, and spleen had the greatest quantities of DEET radioactivity, however, the primary route of DEET elimination and excretion was via urinary excretion of metabolites (48). In addition, estimates of the extent of DEET absorbed across the skin of humans have been made based on urinary excretion of radioactivity (17). Smallwood et al. revealed that urinary DEET concentrations were shown to be positively associated with estimated exposure among volunteers analyzed by a newly developed technique (49). Moreover, rabbits given DEET orally had a lower body weight and a higher kidney weight (50), as results as an early dietary study in albino rats (51). Schoenig et al. (48) reported that when compared to control groups, CD rats treated with DEET had a higher incidence of chronic progressive nephropathy and CD-1 mice had a higher incidence of chronic nephritis. According to *in vitro* human studies, the major pathways of DEET metabolism depend on specific CYP isozymes, by inducing CYP3A, thereby inducing DEET its own metabolism (19). Abou-Donia et al. (52) revealed that DEET decreased the permeability of the blood-brain barrier (BBB) in various brain areas in a repeated dose dermal study in rats, which may attribute to elevate the levels of cyclic adenosine monophosphate (cAMP). This may explain some adverse neurological effects caused by DEET. Although these studies are mainly based on animal studies, they can offer abundant basic evidence to prove and support the possibility of a link between kidney stones and DEET, and encourage us to investigate the probable etiology and pathophysiology of stone formation, which would increase understanding in this area by enabling comparisons of different research.

There are several advantages to this study. First, this is the first thorough study evaluating the relationship between urinary DEET and the risk of kidney stones. Second, because of the multiethnic population from the United States for five consecutive NHANES 2-year cycles (2007–2016), the population is appropriately representative. Third, the indicator we used in this study is quite sensitive because urine is the most often used medium for determining DEET exposure in humans, and because of DEET's fast metabolic rate, both DCBA and DHMB are more sensitive indicators of exposure than DEET itself (53). Therefore, urinary DEET concentration can represent human DEET exposure, so it might be used to produce a complete DEET exposure guide in the future. Inevitably, there are some limitations and deficiencies. First, the data in our study are cross-sectional and the logistic regression cannot prove causal relationships, therefore prospective studies are needed as a next step to confirm their accuracy, and there is little laboratory data concerning human studies. Second, we cannot completely exclude the residual confounding by unmeasured or unknown variables, although we have adjusted for several potential confounders. Third, some asymptomatic kidney stones without physical examination were missed in the database. Fourth, because the NHANES database lacks information on the etiology, size, and treatment history of kidney stones, we could not reveal the association between the different stone types and DEET. In conclusion, albeit the associations are of biological plausibility, the findings should be interpreted with caution and confirmatory longitudinal studies or clinical trials are warranted.

## 5 Conclusion

In summary, DEET exposure is positively associated with the risk of kidney stones, higher content of urine DCBA is more susceptible to kidney stones. Further research is needed to investigate the underlying mechanisms of this association and to guide the prevention and treatment of kidney stones.

## Data availability statement

The original contributions presented in the study are included in the article/Supplementary material, further inquiries can be directed to the corresponding authors.

## Ethics statement

The NHANES data used in this study had already received approval from the NHANES Institutional Review Board (IRB)/NCHS Research Ethics Review Board (ERB), and therefore our study does not require additional approval. The present study was also conducted in accordance with the ethical guidelines of the Declaration of Helsinki.

## Author contributions

CW: conceptualization, data curation, formal analysis, methodology, software, visualization, writing—original draft, and writing—review and editing. JH, ZW, and YH: conceptualization, methodology, and writing—review and editing. WL, MX, and CD: validation and writing—review and editing. XZ, WL, and ZC: conceptualization, funding acquisition, methodology, supervision, and writing—review and editing. ZW: response to comments from reviewers and editors. All authors contributed to the article and approved the submitted version.
